# Dangers of Phosphorus

**Published:** 1883-03

**Authors:** 


### DANGERS OF PHOSPHORUS.

A series of investigations that have been published in several numbers of a German match journal has led to the following general conclusions :

1. The manufacture of matches from white phosphorus, owing to the unavoidable evolution of phosphorus vapors, is fraught with the gravest danger to the health and lives of the wo [illegible] m

2. The vapors of phosphorus, if breathed for a long time, produce general ill-health, under circumstances not yet fully understood, but which are probably to be sought for in the idiosyncrasies of the individual. Usually it takes the local form of necrosis of the maxillary or jaw bone.

3. The necrosis of the jaw, if not relieved in time by an operation, results in death.

4. The injurious constituents of the phosphorus vapors are neither phosphorus acid nor phosphoric acid, but *phosphorous itself*, free and uncombined, which passes into the blood as such, and probably circulates in the blood in the form of vapor, and from the blood acts upon special organs (liver, kidney, heart, stomach and muscles) as well as on the bone tissues.

5. The most dangerous operations in making matches are making up the paste, dipping the splints, drying and packing the matches.

6. The manufacture of matches should only be permitted under the condition that the phosphorus vapors shall be completely excluded from the work-rooms.

7. These conditions can be sufficiently complied with by energetic ventilation, and the use of a safety apparatus.

---

How to Save One Who is Choking.—Dr. J. William White says: "Do not lose an instant. Force the mouth open with the handle of a knife or of a long spoon ; push the thumb and fingers deep down into the throat beyond the root of the tongue, and feel for the foreign body. If the obstruction cannot be grasped, a hair-pin bent into a hook and guided by the left hand will often bring it out. If this fails, get some one to press against the front of the chest or support it against the edge of a table, and strike several hard, quick blows with the open hand on the back, between the shoulder blades. Further treatment must be applied by a physician, who should have been immediately sent for."

